# LZerD Protein-Protein Docking Webserver Enhanced With *de novo* Structure Prediction

**DOI:** 10.3389/fmolb.2021.724947

**Published:** 2021-08-12

**Authors:** Charles Christoffer, Vijay Bharadwaj, Ryan Luu, Daisuke Kihara

**Affiliations:** ^1^ Department of Computer Science, Purdue University, West Lafayette, IN, United States; ^2^ Department of Biological Sciences, Purdue University, West Lafayette, IN, United States

**Keywords:** web server, LZerD, structure modeling, protein bioinformatics, protein-protein docking, protein structure prediction, symmetrical docking

## Abstract

Protein-protein docking is a useful tool for modeling the structures of protein complexes that have yet to be experimentally determined. Understanding the structures of protein complexes is a key component for formulating hypotheses in biophysics regarding the functional mechanisms of complexes. Protein-protein docking is an established technique for cases where the structures of the subunits have been determined. While the number of known structures deposited in the Protein Data Bank is increasing, there are still many cases where the structures of individual proteins that users want to dock are not determined yet. Here, we have integrated the AttentiveDist method for protein structure prediction into our LZerD webserver for protein-protein docking, which enables users to simply submit protein sequences and obtain full-complex atomic models, without having to supply any structure themselves. We have further extended the LZerD docking interface with a symmetrical homodimer mode. The LZerD server is available at https://lzerd.kiharalab.org/.

## Introduction

Protein-protein interactions are key components of many biological processes, and the three-dimensional (3D) structures of the protein-protein complexes thus formed are a crucial resource for reasoning about their molecular functions. Ideally, structures of these complexes would be determined experimentally, through techniques such as X-ray crystallography or cryo-electron microscopy. However, it is possible to instead use computational methods to construct atomic structure models of protein complexes (Aderinwale et al., 2020). This class of methods is called protein-protein docking, and suitably constructed models from docking can be used to reason about how molecular functions are carried out in the living cell, even in the absence of an experimentally determined complex structure (Sanyal et al., 2021). Many protein-protein docking methods exist, such as LZerD (Venkatraman et al., 2009), Multi-LZerD (Esquivel-Rodríguez et al., 2012), ZDOCK (Mintseris et al., 2007), HADDOCK (Dominguez et al., 2003), ClusPro (Kozakov et al., 2017), RosettaDock (Lyskov and Gray, 2008), HEX (Ritchie and Venkatraman, 2010), SwarmDock (Torchala et al., 2013), ATTRACT (de Vries and Zacharias, 2013), and SymmDock (Schneidman-Duhovny et al., 2005). Previously, we released a web-based tool which allows free, easy, installation-free access to LZerD (Christoffer et al., 2021). Users can perform pairwise and multiple chain docking in the LZerD server. Users can also provide additional information in the form of distances of interacting or non-interacting residues to guide docking. The LZerD suite of methods has been ranked at or near the top of all server groups in recent rounds of CAPRI (Lensink et al., 2018; Lensink et al., 2019; Lensink et al., 2020), the blind communitywide assessment of protein docking methods.

Biologists seeking to model a complex computationally may already have structures of individual subunits determined by experiment. However, where no such structures are available, a structure model can be constructed. Single-chain protein structure prediction methods have recently matured, and can often generate models in the absence of clear global template structures (Kryshtafovych et al., 2019). Such methods include our AttentiveDist (Jain et al., 2021), trRosetta (Yang et al., 2020), RaptorX (Xu and Wang, 2019), and QUARK (Zheng et al., 2019). Relative to the top existing servers participating in CASP13 (Kryshtafovych et al., 2019), AttentiveDist showed competitive performance when evaluated on the CASP13 (Kryshtafovych et al., 2019) dataset (Jain et al., 2021).

The underlying methods implemented in the LZerD webserver, LZerD (Christoffer et al., 2021), Multi-LZerD (Esquivel-Rodríguez et al., 2012), and AttentiveDist (Jain et al., 2021), and the ranksum model scoring function (Peterson et al., 2017a; Peterson et al., 2018a; Christoffer et al., 2020) have been rigorously examined in their original papers. In this article, we present the current version of the LZerD webserver, with new functionality of *de novo* prediction of subunit structures by AttentiveDist and applying symmetry constraints for homodimer modeling. We provide step-by-step instruction with examples of modeling in three different scenarios.

## Materials

To perform protein docking, it is best if users have experimentally determined 3D structures. In case the structure is not available, structures can be modelled from the amino acid sequences of the proteins in question. In practice, it is recommended that as much information about the protein structures and their interactions as possible be gathered in advance. For example, there should be evidence that the proteins in question do in fact form a complex, e.g., from a biochemical assay or a biophysical experiment. It is even more desirable that information be known about specific residue interactions or non-interactions. As discussed in later sections, such information can even be provided directly to the server.

## Methods

### AttentiveDist Protein Structure Prediction

For structure modeling of individual proteins, the LZerD server uses AttentiveDist (Jain et al., 2021). If users have 3D structures of individual proteins to dock, they can skip the AttentiveDist step. Here, we give a brief overview the algorithm of AttentiveDist.

In the first stage of AttentiveDist, four multiple sequence alignments (MSAs) with E-value cutoffs of 0.001, 0.1, 1, and 10 are generated using the DeepMSA (Zhang et al., 2020) method, which uses HH-suite (Steinegger et al., 2019) and HMMER (Johnson et al., 2010) to generate MSAs from the UniClust30 (Mirdita et al., 2017), UniRef90 (Suzek et al., 2015), and Metaclust (Steinegger and Soding, 2018) protein sequence databases. A trained neural network is then fed the amino acid types, the PSI-BLAST (Altschul et al., 1997) position-specific scoring matrix, the HMM profile, the secondary structure and solvent-accessible surface area predicted by SPOT-1D (Hanson et al., 2019), rough contacts predicted by CCMPRED (Seemayer et al., 2014), mutual information, and a statistical pairwise potential. The output of this neural network is a prediction of the distribution of residue-residue distances.

Once generated, the predicted distance distribution is converted into full-atom structure models by L-BFGS minimization of predicted short-, medium-, and long-range distance restraints in sequence using PyRosetta (Chaudhury et al., 2010). This minimization results in a pool of models which are then scored by a ranksum method (Christoffer et al., 2020; Peterson et al., 2017a; Peterson et al., 2018a) which aggregates the rankings of the pool by the knowledge-based scoring functions GOAP (Zhou and Skolnick, 2011), DFIRE (Zhou and Zhou, 2002), and ITScorePro (Huang and Zou, 2014), and additionally Rosetta’s REF2015 score (Park et al., 2016), into a single ranking. This ranked pool of models is the end output of AttentiveDist.

Heuristically, a model output by AttentiveDist with a ranksum score ≤20 can be considered particularly confident, and a ranksum gap between models of ≥2R can be considered significant, where R is the ranksum score of the top-ranked model. A ranksum of 20 is the score given when a model is ranked fifth by all component scores and guarantees that at least one component score has ranked the model within the top 5.

Although AttentiveDist was shown to have competitive performance at the time of the development (Jain et al., 2021), there are more recent methods that showed promising performance. Users are also encouraged to try such servers, perhaps those which performed well in recent CASP (Kryshtafovych et al., 2019). Single chain models built by an outside method can be uploaded to the LZerD server.

### Using the AttentiveDist Web Interface

**FIGURE 1:**
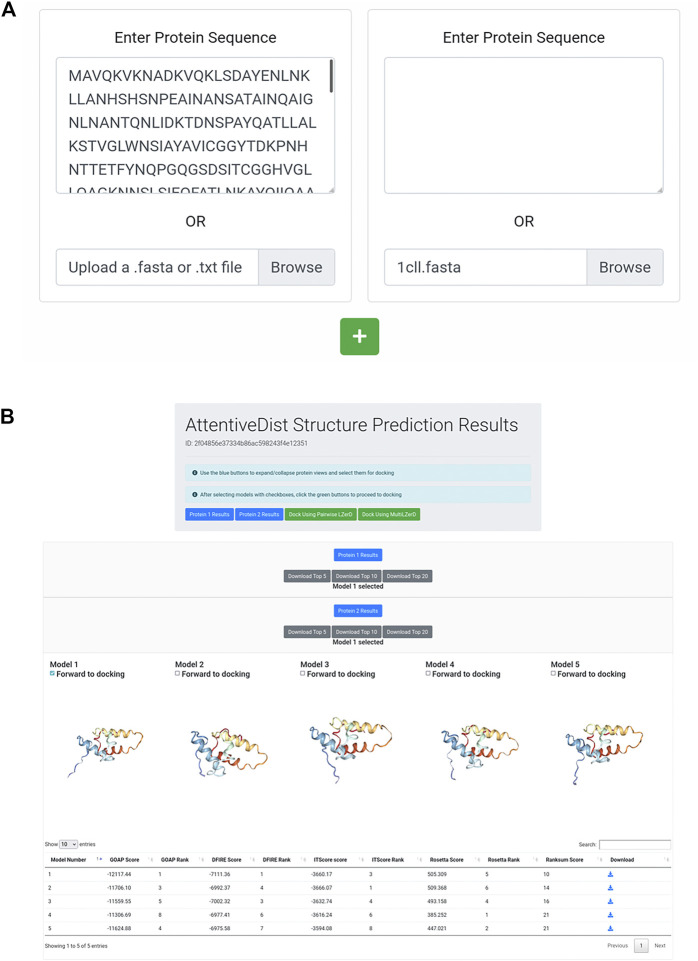
AttentiveDist panels. **(A)** The input page of AttentiveDist where users can input amino acid sequences of subunits to model. By clicking the “+” button, additional sequence submission field will appear. The maximum number of sequences users can submit is six. **(B)** the result panel of AttentiveDist. For each submitted sequence, top five scoring models are visualized. Scores of the five models are shown in a table below the visualization panel. The models are ranked by the ranksum score.

The AttentiveDist web interface (https://lzerd.kiharalab.org/upload/upload_sequence) allows users to submit up to six separate protein sequences for structure prediction at a time. Jobs are limited to six proteins because six is the maximum number of proteins the LZerD server can dock using Multi-LZerD. To submit a sequence, users can simply paste their sequence in FASTA format into the large text box, as shown in Figure 1A. Due to resource constraints, users are limited to 1,000 amino acids per sequence. To submit additional sequences, simply click the “+” button to create a new submission field. Finally, clicking Submit will submit the job. Users can further configure email notification settings, but this is not necessary.

After the prediction job has been submitted and has finished running, users are presented with a results summary page, shown in Figure 1B. This page contains rows of 3D visualizers showing the top five models for each submitted sequence. Below each row of visualizers is a table containing the scoring and ranking data for the output model set. Models can be downloaded in bulk as compressed archives or individually by clicking the appropriately labeled buttons.

From the five models presented for each chain, users need to choose one to perform docking by checking “Forward to docking”. After choosing a model to dock, click either “Dock Using Pairwise LZerD” or “Dock Using Multi-LZerD” to send the models to the docking step. Then, the structure model will be sent to the subsequent step, the protein docking by LZerD. The panel only shows up to the top five models, but up to 20 models can be downloaded locally. If users want, they can examine models within top 20 locally, by using a structure viewer, such as PyMOL (The PyMOL Molecular Graphics System, 2019). Then upload the selected model directly in the input page of the LZerD server.

### LZerD Protein-Protein Docking

The main protein docking engine of the webserver is LZerD for pairwise docking and Multi-LZerD for multiple subunit docking of up to six subunits. Here we briefly explain the algorithms of LZerD and Multi-LZerD.

LZerD takes two structures provided by users (conventionally referred to as receptor and ligand in descending order of size) as input and samples of all possible interaction interface regions and interaction angles exhaustively. If a putative complex structure contains excessive steric violations at the interface, has too small an interaction area, or has low shape complementarity at the interface region, that model is rejected. In LZerD, a protein structure is represented by a molecular surface, which is segmented into overlapping local surface regions. Each local surface region is represented both by a mathematical moment-based shape descriptor called a 3D Zernike descriptor (3DZD) (Kihara et al., 2011). 3DZDs are rotation-invariant, which allows fast, alignment-free computation of shape complementarity, and also allows a soft representation of surface that is robust to a certain degree of conformational change induced by the interaction. This advantage extends to tolerance of small modeling inaccuracies. The conformational space is searched by the geometric hashing algorithm. If constraints of residue-residue distances, interface residues, or symmetry tolerance have been provided, models violating the constraints are rejected.

LZerD generates tens of thousands of docking models. After clustering (by default at an RMSD cutoff of 4.0 Å), which generally culls the model pool to a few thousand to a few tens of thousands, the complex models are then scored by a ranksum method (Peterson et al., 2017a; Peterson et al., 2018a; Christoffer et al., 2020) which aggregates the rankings of the pool by the knowledge-based scoring functions GOAP (Zhou and Skolnick, 2011), DFIRE (Zhou and Zhou, 2002), and ITScorePro (Huang and Zou, 2014). These three scoring functions essentially check if atom interactions in a model have distances and angles that agree with those observed in experimentally determined protein structures overall. If a model is consistently ranked as the top by all the component scores, then the ranksum of the model will be low, e.g. 3 if a model is ranked 1 by all component scores. Ranksum has been shown to perform very well in docking model ranking in CAPRI protein docking assessments (Lensink et al., 2019; Lensink et al., 2020).

Multi-LZerD takes three to six protein structures and builds them into a complex. It first runs pairwise LZerD for each pair of subunits to generate a pool of pairwise docking models. Then, it subsequently selects pairwise models and assembles into full subunit models. Combinations of pairwise models are iteratively optimized by a genetic algorithm. In Multi-LZerD, models are selected with a molecular mechanics force field with terms reweighted specifically for protein docking.

#### Homodimer Docking (C_2_ Symmetry)

Symmetrical protein complexes are often observed in nature (Levy et al., 2006). Cyclic symmetry of general order n isusually referred to as $C_{n}$ symmetry. The LZerD server supports docking with C_2_ symmetry, i.e., homodimers, in the current version of the server because homodimers are quite common. A measure of the C_2_ symmetricalness of a model can be constructed by transforming the atomic coordinates of the subunit by applying the rigid body transformation from docking twice and calculating the root-mean-square deviation (RMSD) to the original coordinates. If the model is perfectly symmetrical, this RMSD will be 0 Å. For the $C_{2}$ symmetry constraint functionality provided in this webserver, this RMSD is cut off at 5.0 Å, and models exceeding it in symmetry mode are discarded.

### Using the Docking Web Interface

**FIGURE 2:**
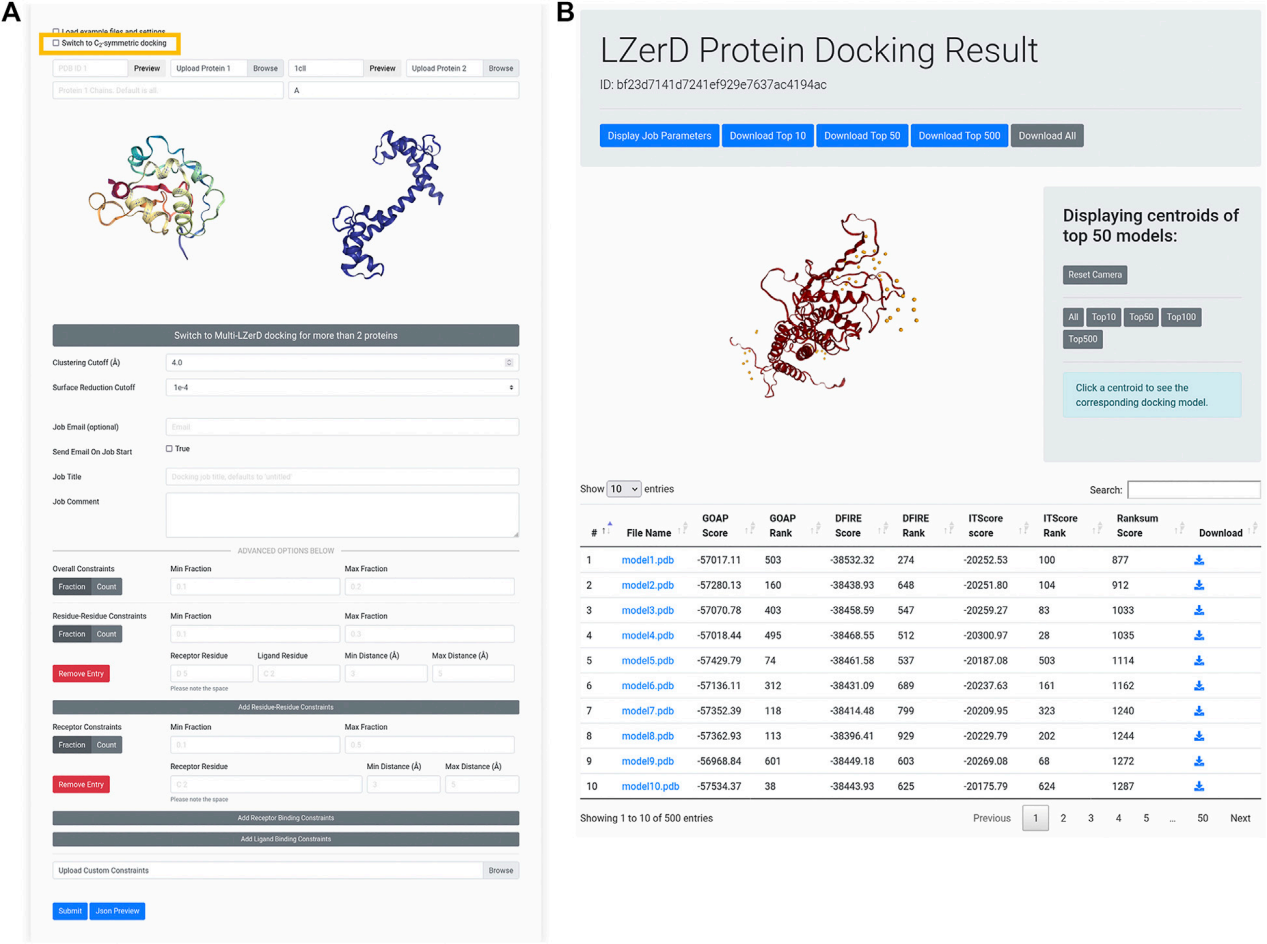
Submitting and interpreting protein docking job. **(A)** the job submission page. The figure represents a situation that a structure model of Protein 1 is transferred from AttentiveDist and the structure for Protein 2 will be fetched from PDB. The check box for performing C_2_ Symmetry docking is highlighted by rectangle in yellow. **(B)** docking results page. On the top of the panel, a distribution of centroids of the docked poses of the ligand structures are indicated with spheres. By clicking a sphere, the docked structure of the pose will be presented.

Figure 2 shows the interface for submitting individual protein structures for docking. Once users have forwarded subunit structures from the AttentiveDist web interface, the structures appear in the input panel as shown in Figure 2A. Alternatively, users can upload structures from their local disk or fetch them directly from the Protein Data Bank (PDB) (Berman et al., 2000) *via* the upload widget. It is also possible to model one of the subunit structures by AttentiveDist and dock it with a structure from PDB. Clicking the Submit button will start the docking computation.

This will run LZerD with the recommended default settings and without constraints. If users prefer, they can directly change parameters: the clustering RMSD cutoff, which controls the redundancy of the output model pool, as well as the surface reduction cutoff, which in part controls how finely the conformational space is sampled. If users are modeling a symmetrical homodimer, they can select the checkbox to model with a $C_{2}$ symmetry constraint, which excludes parts of the conformational space greater than 5 Å RMSD from perfect symmetry. Users can further optionally supply an email for receiving job notifications, whether to receive a notification when a job begins running, a job title, and a job comment describing what the job is modeling.

Below, in the advanced options section, users can specify distance constraints for specific residue-residue interactions or residue-subunit interactions. Distances used for these constraints should ideally come from experiment, but can also come from computational predictions (La et al., 2013, La and Kihara, 2012). All distance constraints specify an allowed range for the closest heavy (non-hydrogen) atoms between two selections of atoms. For residue-residue constraints, the selections are the atoms belonging to the two specified residues. For receptor binding site constraints, the selections are the specified receptor residue and the entire ligand subunit. For ligand binding site constraints, the selections are the specified ligand residue and the entire receptor subunit. If a particular pair of residues should be in contact, a user could for example specify a distance range of 0–5 Å, or perhaps 0–8 Å or even broader depending on what available data from experiment might indicate. To specify that two residues should not interact, a user could for example specify an exclusionary minimum distance such as 15 Å. The same logic can be applied to receptor binding site and ligand binding site constraints. To control the number of constraints that must be satisfied, users can set the min/max fraction fields, which directly specify what proportion of the distance constraints should be satisfied. A toggle is available for users to switch from specifying the proportion to specifying the actual numbers of constraints that are allowed to be satisfied.

The submission process is essentially the same for Multi-LZerD. Only the difference is, naturally, to specify three or more (up to six) subunit structures to assemble. To switch to Multi-LZerD, click the large gray button of “Switch to Multi-LZerD docking for more than 2 proteins”.

After the docking job has been submitted and has finished running, the user is presented with a results summary page (Figure 2B). This page contains a 3D visualizer showing the distribution of ligand centroids of the top-scoring docked models, with a user selectable top-k threshold. Below the visualizer is a table containing the scoring and ranking data for the output model set. As default, models are ranked by the ranksum score. Users can choose another score to sort the models by clicking an arrow of the preferred score. Users can click on a model to display its 3D structure. Models belonging to particular centroids can be displayed by clicking on those centroids. Docked models can be downloaded in bulk as compressed archives or individually by clicking the appropriately labeled buttons.

For more information about the job submission steps and interpretation of results page, users are encouraged to refer to the instructions on the LZerD web server. From the top bar, the information is available from the “About” pull-down menu.

## Results

We discuss three case studies of docking modeling in different scenarios. The first case is the regular pairwise protein docking. The second case is homodimer docking, while the last case is docking with structure models built through the AttentiveDist pipeline.

### Case Study: Regular LZerD Docking of Human IL23-IL23R

**FIGURE 3:**
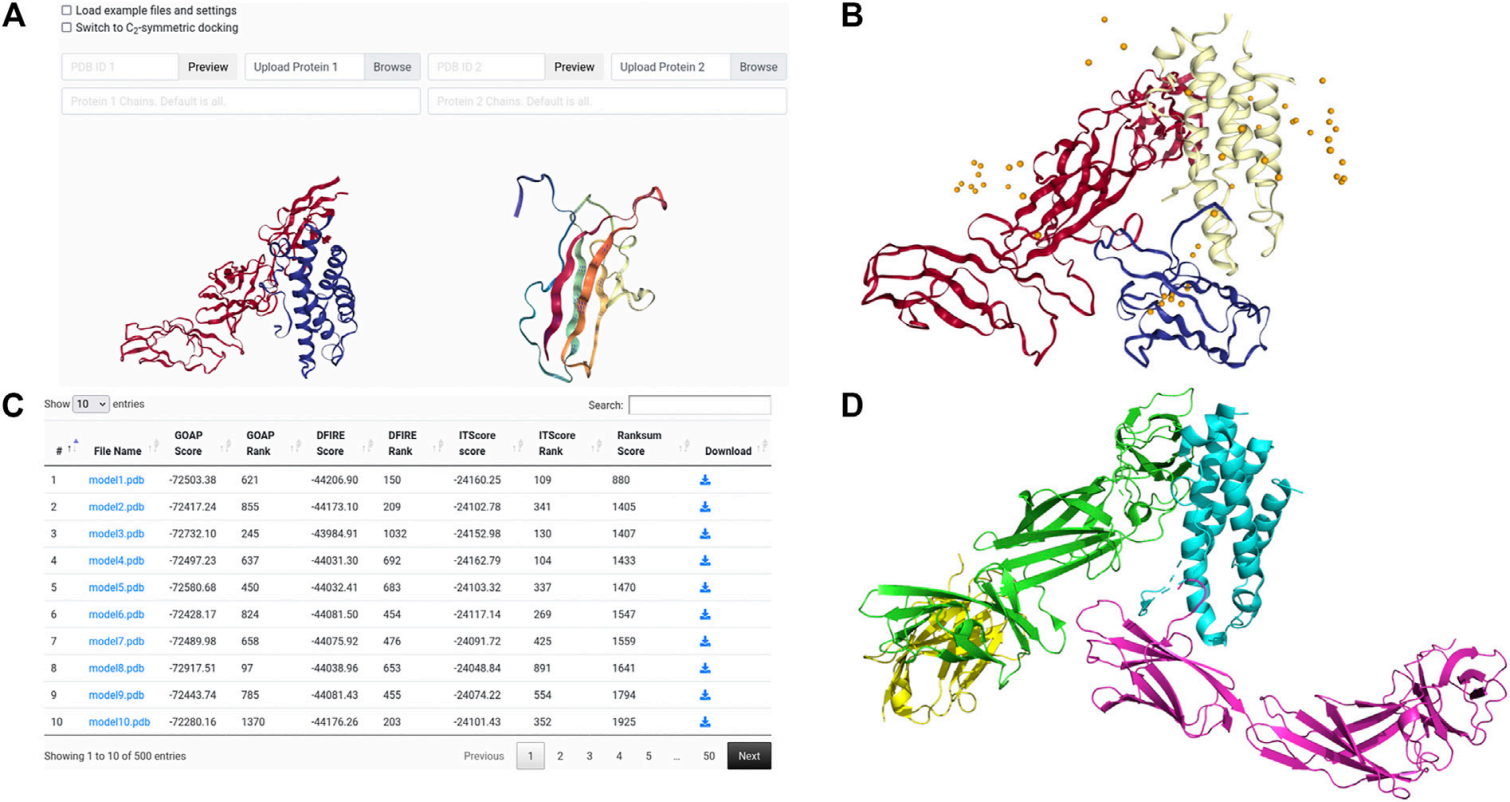
Input and results for unconstrained docking of IL23-IL23R. **(A)** the input for unconstrained LZerD. The model of IL23 was uploaded as the receptor on the left, while the model of IL23R was uploaded as the ligand on the right. The chain ID selection fields are blank since we want to use all the chains. This docking run was done without constraints, so the entire constraints section is empty. **(B)** the results of unconstrained docking. IL23 is shown in red, while IL23R is shown in blue. The cartoon structure shown is the top-10 model, which has an $f_{nat}$ of 0.28, an I-RMSD of 4.4 Å, and an L-RMSD of 7.8 Å, which is of acceptable CAPRI quality. The distribution of the top 50 ligand centroids is indicated by the orange spheres. **(C)** The table of model scoring information for this docking run. The ranksum score, which is used to finally rank the models, is on the right. **(D)** the native structure of this complex, PDB 5MZV. The non-interacting domains of IL23R and a nanobody bound to IL23 are included in the view.

The first case study provides an example of basic pairwise protein docking modeling. During CAPRI Round 39, a complex of a human cytokine heterodimer IL23 with a human IL23R monomer was given for prediction as target T122. CAPRI is a blind experiment where the structure of the complex to be modeled is not known to the predictors until well after all predictions have been submitted. In this example we show a similar result to the LZerD server group’s modeling performance on this target (Christoffer et al., 2020). As the LZerD server group did during CAPRI, here we used an unbound structure from X-ray crystallography provided by the organizers for IL23, which has a root-mean-square deviation (RMSD) of 1.7 Å to the native T122 structure. For the ligand, we used a model of IL23R generated by template-based modeling with MODELLER (Webb and Sali, 2021) which has been truncated to the actual interacting domain, which has an RMSD of 3.2 Å to the native T122 structure. As suggested by the low RMSD, the template-based modeling was reasonably successful at predicting the structure of IL23R. For the docking, we set no constraints. In Figure 3, we show the results of this LZerD job. The top-10 model shown is acceptable under the CAPRI evaluation criteria, with a ligand RMSD (L-RMSD) of 7.8 Å and a fraction of native contacts (*f*_nat_) of 0.28 relative to the native structure PDB 5MZV shown in Figure 3D. Although this result is good, the richness of the result set can be increased by adding constraints, as discussed in the corresponding case study section in a previous paper (Christoffer et al., 2021). As seen by the centroid distribution in Figure 3B, the ligand models are not concentrated at the native interaction site.

### Case Study: LZerD Docking of Bacterial Aminoglycoside 2′-N-Acetyltransferase Homodimer With $C_{2}$ Symmetry Constraint

**FIGURE 4:**
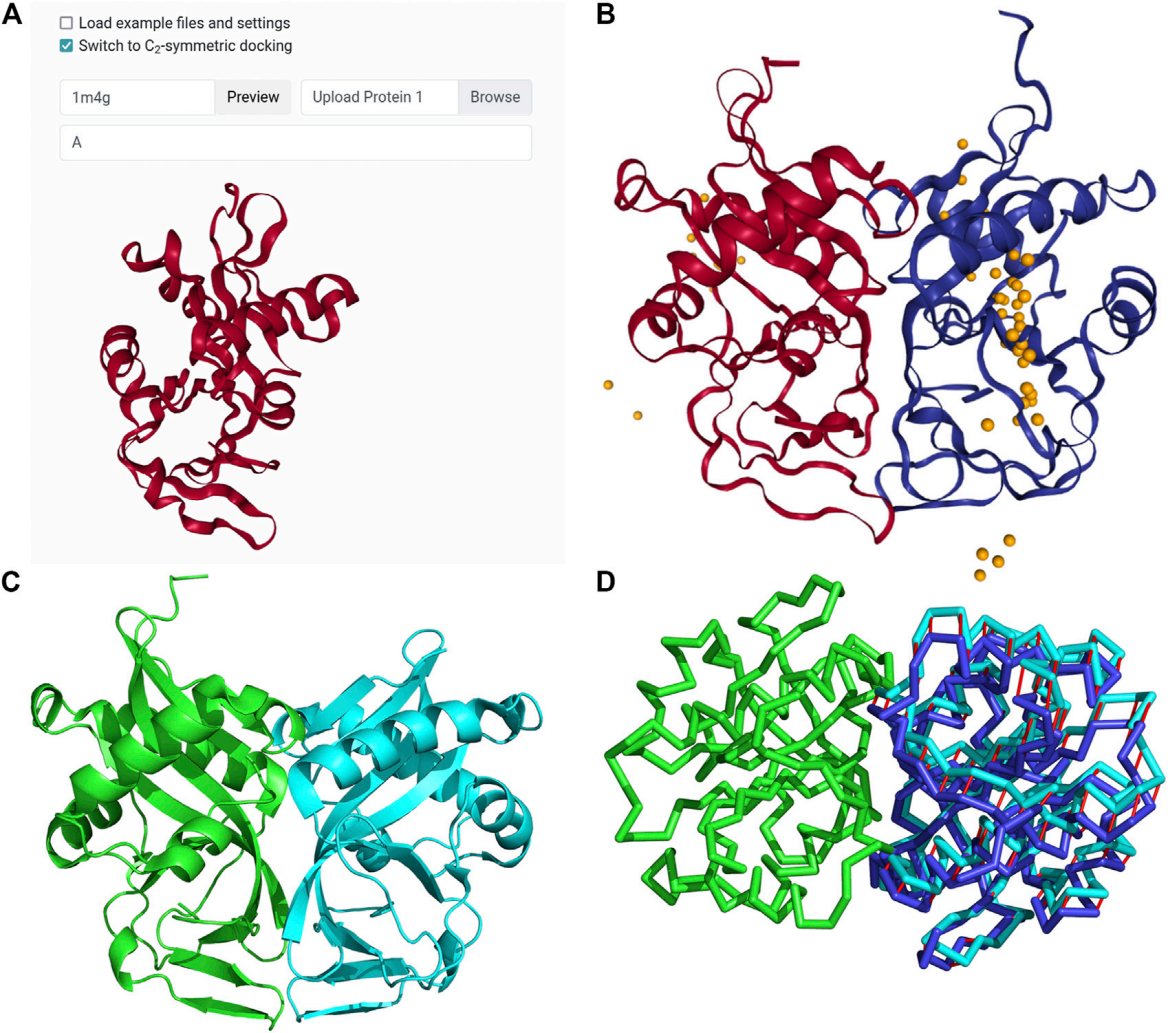
Input and results for C_2_ symmetrical docking of bacterial AAC (2′). **(A)** The input for symmetrical LZerD. The subunit was uploaded by specifying the PDB ID 1M4G in the input field to fetch the structure from the PDB. A single chain is extracted from this structure by specifying “A” in the chain ID selection field. **(B)** The results of C_2_ symmetrical docking. The receptor and ligand are shown in red and blue respectively in the top-1 model conformation and are of course structurally identical. This model has an $f_{nat}$ of 0.88, an I-RMSD of 0.95 Å, and an L-RMSD of 1.9 Å. The distribution of the top 50 ligand centroids is indicated by the orange spheres. All output models satisfy the 5.0 Å symmetricalness criterion. **(C)** the native structure of this complex, both chains of PDB 1M4G. Note that some N-terminal residues of chain B (cyan) are not resolved relative to chain A (green). In fact, considering all common atoms between the two native chains, they differ by 0.8 Å RMSD. **(D)** Visualization of the symmetricalness criterion’s satisfaction in the top-1 model viewed along the C_2_ symmetry axis. The subunits are shown here in Cα trace representation. Green: the receptor; cyan: the correct ligand conformation; blue: the top-1 ligand conformation; red: the deviations of the ligand from the correct conformation. This model satisfies the symmetricalness criterion with an RMSD of 2.2 Å.

This is an example of modeling a homodimer complex with the newly implemented homodimer constraint. The complex used is aminoglycoside 2′-N-acetyltransferase (AAC (2′)) from *M. tuberculosis*, which appears to directly relate to the drug resistance of the organism (Vetting et al., 2002). This example was used in a work by Ritchie and Grudnin on symmetrical protein docking (Ritchie and Grudinin, 2016). To create the LZerD job, we used the PDB ID input method to specify that 1M4G should be fetched from the PDB. Then, we used the Protein 1 Chains field to specify that only chain A should be considered. Finally, we selected the checkbox to switch to symmetrical docking (Figure 4A) and clicked Submit. In the referenced work (Ritchie and Grudinin, 2016), a docked model was considered a hit if it had an RMSD within 10 Å of the native structure. According to their paper (Ritchie and Grudinin, 2016), M-ZDOCK and SymmDock found no hits within the top 10, while SAM’s top-1 model was a hit with RMSD 1.82 Å. LZerD in $C_{2}$ symmetry mode’s top-1 model, shown in Figure 4B, was a hit, with an even lower RMSD of 0.94 Å. By the CAPRI criteria, this model is of high quality, with an $f_{nat}$ of 0.88, an I-RMSD of 0.95 Å, and an L-RMSD of 1.9 Å. Figure 4C shows the native structure. In Figure 4D, we examined the symmetry of our model. Our model (blue) has an RMSD of 2.2 Å to the correct pose (cyan) that is located at the perfect symmetrical position.

### Case Study: LZerD Docking of Designed colEdes3:Imdes3 With Site-Directed Mutagenesis Data

**FIGURE 5:**
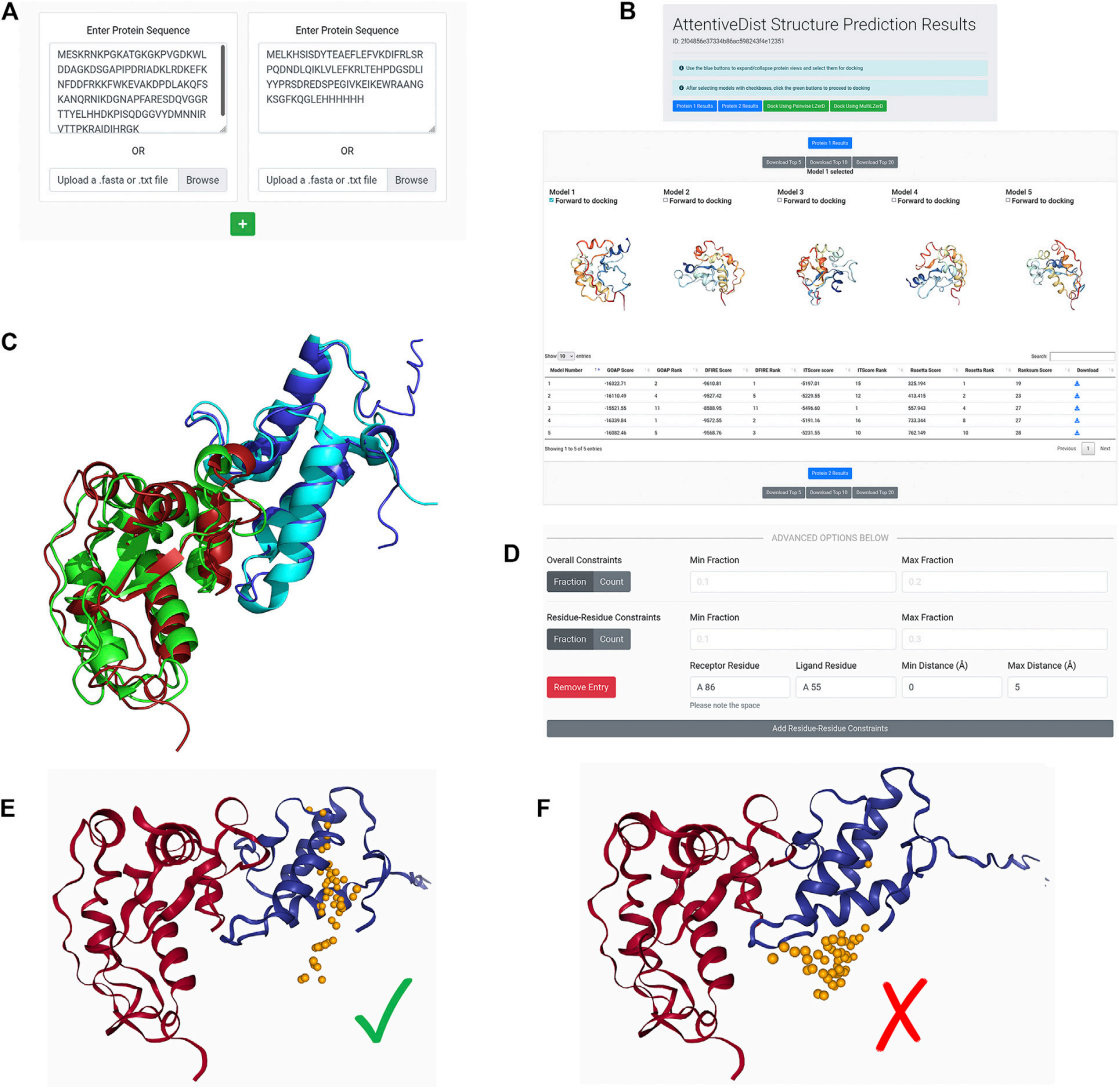
De novo subunit modeling and LZerD docking of colEdes3:Imdes3. **(A)** The input for *de novo* modeling of the subunits with AttentiveDist. The sequences were pasted into the input fields, but users can alternatively upload FASTA files. **(B)** The results of *de novo* structure prediction. Both subunits are available from this page, and colEdes3 is currently selected for display. Users can download models individually or in bulk and can forward models to LZerD by selecting the checkboxes and clicking the LZerD or Multi-LZerD button. The scoring table appears below the 3D models. **(C)** The top-1 AttentiveDist models superimposed to the native structure (green and cyan; PDB ID: 6ERE). The top-1 models for colEdes3 (red) and Imdes3 (blue) have RMSDs of 2.5 Å and 2.0 Å. **(D)** The constraint used for LZerD docking. Here, Phe68 of colEdes3 was constrained to be in contact with Tyr55 of Imdes3 by specifying a distance cutoff of 5.0 Å. **(E)** Results of constrained LZerD. Model 5 is shown, and is of acceptable quality with an of 0.32, an I-RMSD of 3.9 Å, and an L-RMSD of 10.7 Å. As indicated by the centroid distribution, the docking search has been focused around the binding site by the constraint. (**F)** Results of unconstrained LZerD. Model 13 is shown, but is not of acceptable quality, with an $f_{nat}$of 0.36, an I-RMSD of 4.2 Å, and an L-RMSD of 13.1 Å. This I-RMSD barely missed the CAPRI threshold for acceptable quality. Without any constraint, the docking for this input does not produce acceptable models in the top 10. As indicated by the centroid distribution, the docked models are largely preferring a different site.

In this last example, we started from the sequence of individual proteins to model their tertiary structures by AttentiveDist, which were then docked to obtain complex models. In CAPRI round 43, a redesigned version of the *E. coli* colicin-E2:DNase-Im2 complex was presented as the target T133. The structure of this complex has since been released, and is available as an entry in PDB, 6ERE. The designed sequences of the first assembly, i.e. chains B and C, can be taken from the PDB entry page (https://www.rcsb.org/structure/6ERE). We modeled this complex from the sequence information without using any template structures. We clicked Upload Protein Sequences to bring up the AttentiveDist submission form and pasted the colEdes3 sequence. Then, we clicked the green plus button to add another sequence input field and pasted the Imdes3 sequence there. The filled submission form is shown in Figure 5A. Figure 5B shows the result of the single-chain modeling. From this panel, we selected the first model for each subunit as shown in Figure 5B, and then clicked Dock Using Pairwise LZerD to forward both models to the LZerD submission page. The top-1 models for colEdes3 and Imdes3 have RMSDs of 2.5 Å and 2.0 Å to the native structure, respectively, and can be seen superimposed in Figure 5C.

From the site-directed mutagenesis experiments, we knew that Tyr55 of DNase-Im2 is a hotspot residue (Netzer et al., 2018). Further, this residue is conserved by the designed sequence of Imdes3 (Wojdyla et al., 2012). The same is true for Phe86 of colicin-E2, a key specificity site. Thus, to focus the docking, we integrated this information into our docking job. To accomplish this, we clicked Add Residue-Residue Constraints and create a constraint between receptor residue “A 86” indicating chain A residue sequence number 86, and ligand residue “A 55”, with minimum distance 0 Å and maximum distance 5 Å (Figure 5D). This configuration tells the LZerD server that Phe86 of colEdes3 and Tyr55 of Imdes3 should be in direct contact with each other. We left the Min/Max Fraction fields blank since we were only specifying one single constraint.

On the results page, shown in Figure 5E, the distribution of ligand centroids about Phe86 of colEdes3 is clearly visible. The top-10 model pool contains two acceptable models: model 5 is acceptable with an $f_{nat}$ of 0.32, an I-RMSD of 3.9 Å, and an L-RMSD of 10.7 Å; model 8 is acceptable with an $f_{nat}$ of 0.40, an I-RMSD of 3.8 Å, and an L-RMSD of 11.6 Å. In this example, the residue constraints were effective in guiding the docking. To compare, in Figure 5F, we show docking results when the constraints were not provided. As shown, without the constraints the ligand was attracted at an incorrect place, which made a larger interface between the two proteins.

This example demonstrates several qualities of the pipeline components and qualities of the pipeline as a whole. As shown quantitatively by the metrics and visually in Figure 5C, AttentiveDist was capable of accurately modeling the structures of individual protein chains. As shown by the unconstrained result, the correct interface was sampled in docking with no template or extra interaction information, although a model of acceptable CAPRI quality was not ranked at the top. As shown quantitatively by the metrics and visually in Figure 5E, the barest of residue-residue interaction information was sufficient to overcome this and produce acceptable models among the top ranks.

## Discussion

With the upgraded LZerD web interface, biologists can conveniently construct protein complex models that they can use to reason about the interactions in their system. Information about the system can be integrated in the form of geometric distance and symmetry constraints. Now, even without known or template-modeled subunit structures, users can generate *de novo* predictions of subunit structures and dock them with the click of a button. Future development of the LZerD web platform is expected to include modeling complexes with intrinsically disordered proteins using IDP-LZerD (Peterson et al., 2017b), as well as modeling the assembly order of multimeric complexes using Path-LZerD (Peterson et al., 2018b).

## Data Availability

The original contributions presented in the study are included in the article/supplementary material, further inquiries can be directed to the corresponding author.
